# A Simple-To-Use Nomogram for Predicting Early Death in Metastatic Renal Cell Carcinoma: A Population-Based Study

**DOI:** 10.3389/fsurg.2022.871577

**Published:** 2022-03-22

**Authors:** Tao Chen, Xiangpeng Zhan, Junfu Du, Xiaoqiang Liu, Wen Deng, Shuaishuai Zhao, Ming Jiang, Yunqiang Xiong, Xiaohai Zhang, Luyao Chen, Bin Fu

**Affiliations:** ^1^Department of Urology, The First Affiliated Hospital of Nanchang University, Nanchang, China; ^2^Department of Urology, Wuning People's Hospital, Jiujiang, China; ^3^Jiangxi Institute of Urology, Nanchang, China

**Keywords:** renal cell carcinoma, SEER database, early death, prognosis, nomograms

## Abstract

**Background:**

Metastatic renal cell carcinoma (mRCC) is usually considered to have a poor prognosis, which has a high risk of early death (≤3 months). Our aim was to developed a predictive nomogram for early death of mRCC.

**Methods:**

The SEER database was accessed to obtain the related information of 6,005 mRCC patients between 2010 and 2015. They were randomly divided into primary cohort and validation cohort in radio of 7:3. The optimal cut-off point regarding age at diagnosis and tumor size were identified by the X-tile analysis. Univariate and multivariate logistic regression models were applied to determine significant independent risk factors contributed to early death. A practical nomogram was constructed and then verified by using calibration plots, receiver operating characteristics (ROCs) curve, and decision curve analysis (DCA).

**Results:**

There were 6,005 patients with mRCC included in the predictive model, where 1,816 patients went through early death (death within ≤3 months of diagnosis), and among them 1,687 patients died of mRCC. Based on 11 significant risk factors, including age, grade, *N*-stage, histologic type, metastatic sites (bone, lung, liver and brain) and treatments (surgery, radiation, and chemotherapy), a practical nomogram was developed. The model's excellent effectiveness, discrimination and clinical practicality were proved by the AUC value, calibration plots and DCA, respectively.

**Conclusions:**

The nomogram may play a major part in distinguishing the early death of mRCC patients, which can assist clinicians in individualized medicine.

## Introduction

Renal cell carcinoma (RCC) is derived from the abnormal differentiation of renal tubular epithelial cells, accounting for ~ 2–3% of adult malignant tumors (1). In the past 30 years, the incidence of RCC morbidity has been rising continuously (2). Although with the progress of diagnostic and surgical techniques, early stage RCC can be detected and resected in time, a growing number of patients are diagnosed with distant metastasis at the beginning of diagnosis (3). In addition, even nephrectomy is completed, 20% of patients will re-emerge and progress to mRCC (4). The prognosis of patients with metastatic renal cell carcinoma is strikingly poor and only 12% survive beyond 5 years of diagnosis (5). Due to the clinical application of molecular targeted therapies, including VEGFR, mTORC1, FGFR inhibition and anti PD-1/PD-L1 immune checkpoint inhibitors, great progress has been made on the treatment of mRCC (6). However, patients with mRCC are still vulnerable to premature death, whose reason remains to be not solved. Exploring the risk factors related to early death is instrumental for clinicians in identifying the high-risk population of early death and formulating individualized treatment to reduce the incidence of early death. However, so far, there is no in-depth study on the mortality rate of premature death in mRCC patients. Consequently, it is greatly necessary to establish a simple-to-use model to determine the risk factors leading to early death of mRCC.

The nomogram, as a useful statistical model, can integrate relevant factors to predict the individual oncologic prognosis (7). Nomograms have been extensively applied to assist medical doctors in formulating treatment plans and evaluate the prognosis of all kinds of cancers. National Comprehensive Cancer Network guidelines have introduced nomograms with excellent performance (8).

Here, our study data originated from the Surveillance, Epidemiology, and End Results (SEER) database, which is an authoritative cancer population registry in the United States collecting about 34.6% of the cancer incidence rate and survival data of the American Cancer Registry. We obtained the clinical and pathological features of mRCC and recognized risk factors to establish a practical nomogram for predicting its early death.

## Materials and Methods

### Patient Cohorts

**Figure 1:**
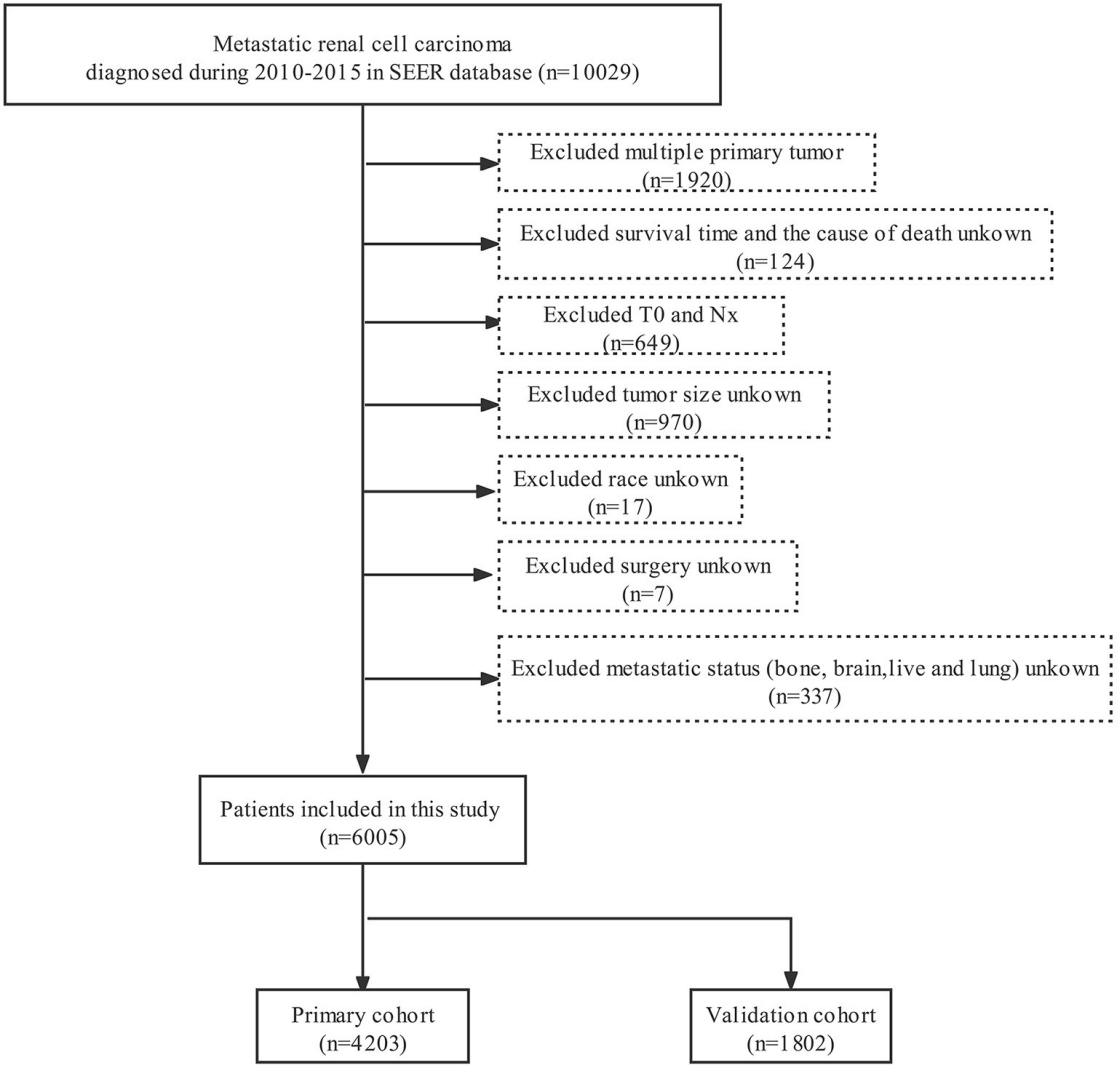
Flowchart for selection of metastatic renal cell carcinoma.

SEER^*^Stat software (Version 8.3.6) was applied to extracted data including demographic and clinical characteristics. In our study, patients with mRCC in the SEER database registered from 2010 to 2015 were selected. Patients enrolled in our study met the following inclusion criteria: (a) the site code was C64.9. (b) the histological codes were 8,050/3, 8,260/3, 8,310/3, 8,317/3, 8,318/3, and 8,319/3. The following criteria should be excluded: (a) unknown/missing cause of death and survival month. (b) incomplete clinicopathological and demographic information including race, tumor size, *N*-stage, metastatic status (bone, brain, liver and lung) and *T*0-stage. (c) uncertain treatment information including surgery, radiotherapy or chemotherapy. Figure 1 shows the detailed screening procedure. According to previous studies, we defined early death as death within 3 months since the first diagnosis (9, 10).

**Figure 2:**
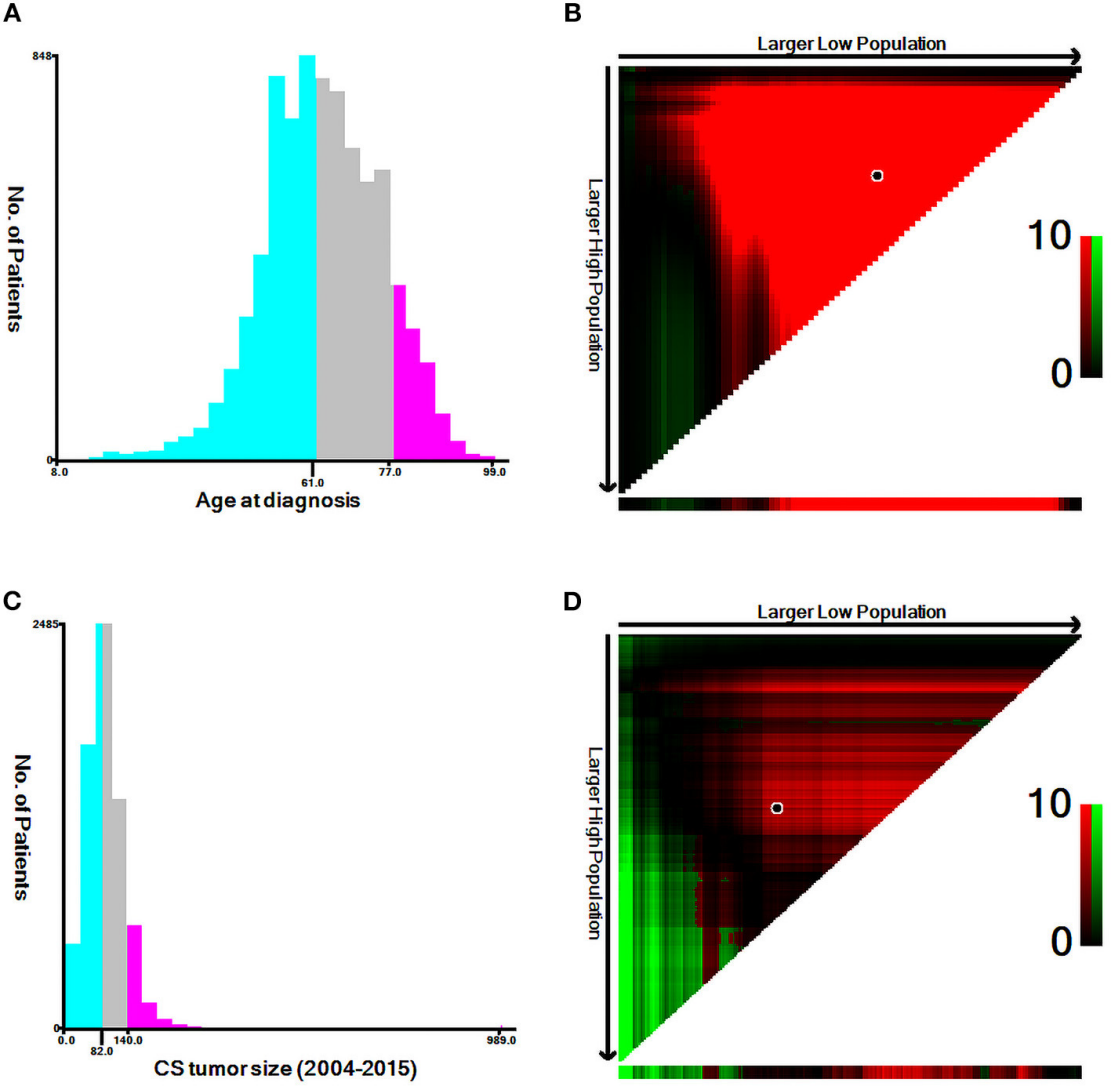
Estimation of the appropriate cut-off value for the age and tumor size by using X-tile analysis. **(A,B)** The optimal cut-off values of age were 61 and 78 years. **(C,D)** The optimal cut-off values of tumor size were 82 and 141 mm.

The following variables were obtained: diagnostic age, gender, ethnicity, histologic type, tumor size, grade, *T*-stage (AJCC 7^th^ version), *N*-stage (AJCC 7^th^ version), bone, lung, liver, and brain metastasis, surgery, radiotherapy, chemotherapy, cause of death, survival months. With respect to diagnostic age and tumor size, we used the X-tile software to calculate the optimal cutoff point (Figure 2).

### Nomogram Construction and Statistical Analyses

The baseline characteristics of the included population were depicted in the form of number and percentage (*n*, %). Univariate and multivariate logistic regression models were applied to calculate odds ratios (OR) and 95% confidence intervals (CI) to determine independent risk variables for the early death of mRCC. Then we used the connected risk factors to establish a simple-to-use nomogram for predicting the early death of mRCC. The calibration and discrimination of the model were assessed by the C-index, receiver operating characteristic curve, the area under the curve (AUC) (11) and calibration plots (12). In addition, we further evaluated the clinical utility by decision curve analysis (DCA) (13). All statistical analysis was completed by using SPSS (version 24.0; SPSS, Inc.), X-tile software, packages (rms, pROC, and rmda) in R software version 4.1.2. We considered two-tailed *p*-value <0.01 as statistically significance.

## Results

### Demographic and Clinical Characteristics

6,005 patients with mRCC were included from the SEER database according the inclusion and exclusion criteria, where 1,816 patients went through early death due to all-cause death, and 1,687 patients died early from mRCC. We divided 4,203 patients into the primary dataset and 1,802 patients into the validation dataset. Among these patients who experienced premature death, most of them were male (66.5%), white (82.5%) and between the age of 62 and 77 years (43.6%). The most common histologic type related to early death was clear cell renal cell carcinoma (27.3%) except for unknown histological classification and most tumor size was focused on < 14 cm (89.2%). The early mortality of poorly differentiated / undifferentiated mRCC and well differentiated / medium mRCC were 23.3 and 5.5%, respectively. The bulk of tumors were *T*3 (30.8%) and *N*0 (56.9%). Concerning treatment, most of them were not treated surgically (83.8%), without radiotherapy (76.2%) and without chemotherapy (73.1%). In addition, at diagnosis, 66.7, 41.2, 15.9 and 30.8% of the patients who went through premature death had lung, bone, brain and liver metastases, respectively. The cohort differences between early death and no early death group were exhibited in Table 1. The detailed characteristics of the mRCC patients in the primary cohort and validation cohort were shown in Table 2.

**Table 1:** Early death or without early death events in patients with mRCC.

**Characteristic**	**SEER cohort (*****n*** **=** **6,005)**		
	**Early death (%)**	**No early death (%)**	***P*-value**
All	1,816 (30.2)	4,189 (69.8)	
**Age**			<0.001*
≤ 61	575 (31.7)	2,052 (49.0)	
62–77	791 (43.6)	1,715 (40.9)	
≥78	450 (24.8)	422 (10.1)	
**Sex**			**0.007**
Male	1,207 (66.5)	2,931 (70.0)	
Female	609 (33.5)	1,258 (30.0)	
**Race**			**0.002**
White	1,499 (82.5)	3,438 (82.1)	
Black	204 (11.2)	397 (9.5)	
Others	113 (6.2)	354 (8.5)	
**Histologic type**			<0.001*
ccRCC	495 (27.3)	2,434 (58.1)	
pRCC	66 (3.6)	202 (4.8)	
sRCC	129 (7.1)	208 (5.0)	
chRCC	4 (0.2)	37 (0.9)	
cdRCC	15 (0.8)	28 (0.7)	
RCC	1,107 (61.0)	1,280 (30.6)	
**Size**			0.424
≤ 82	843 (46.4)	1,929 (46.0)	
83–140	776 (42.7)	1,846 (44.1)	
≥141	197 (10.8)	414 (9.9)	
**Grade**			<0.001*
GI-II	100 (5.5)	582 (13.9)	
GIII-IV	423 (23.3)	1,777 (42.4)	
Unknown	1,293 (71.2)	1,830 (43.7)	
***T*** **-stage**			<0.001*
*T*1	392 (21.6)	797 (19.0)	
*T*2	391 (21.5)	840 (20.1)	
*T*3	559 (30.8)	1,979 (47.2)	
*T*4	250 (13.8)	355 (8.5)	
TX	224 (12.3)	218 (5.2)	
***N*** **-stage**			<0.001*
*N*0	1,033 (56.9)	2,901 (69.3)	
*N*1	432 (23.8)	707 (16.9)	
*N*2	351 (19.3)	581 (13.9)	
**Bone metastasis**			**0.010**
No	1,067 (58.8)	2,609 (62.3)	
Yes	749 (41.2)	1,580 (37.7)	
**Lung metastasis**			<0.001*
No	605 (33.3)	1,689 (40.3)	
Yes	1,211 (66.7)	2,500 (59.7)	
**Liver metastasis**			<0.001*
No	1,256 (69.2)	3,589 (85.7)	
Yes	560 (30.8)	600 (14.3)	
**Brain metastasis**			<0.001*
No	1,527 (84.1)	3,772 (90.0)	
Yes	289 (15.9)	417 (10.0)	
**Surgery**			<0.001*
No	1,521 (83.8)	1,862 (44.4)	
Yes	295 (16.2)	2,327 (55.6)	
**Radiotherapy**			<0.001*
No	1,383 (76.2)	2,837 (67.7)	
Yes	433 (23.8)	1,352 (32.3)	
**Chemotherapy**			<0.001*
No	1,328 (73.1)	1,590 (38.0)	
Yes	488 (26.9)	2,599 (62.0)	
**Cause of death**			<0.001*
mRCC	1,687 (92.9)	2,833 (67.6)	
Other cause	129 (7.1)	1,356 (32.4)	

*ccRCC, clear cell renal cell carcinoma; pRCC, papillary renal cell carcinoma; chRCC, chromophobe renal cell carcinoma; sRCC, sarcomatoid renal cell carcinoma; cdRCC, collecting duct renal cell carcinoma; RCC, renal cell carcinoma*.

*The bold values mean statistically significance (p < 0.01)*.

**Table 2:** Characteristics with mRCC patients in primary and validation cohort.

**Characteristics**	**All patients (%)**	**Primary cohort (%)**	**Validation cohort (%)**
All	6,005 (100.0)	4,203 (70.0)	1,802 (30.0)
**Age**			
≤ 61	2,627 (43.7)	1,821 (43.3)	806 (44.7)
62–77	2,506 (41.7)	1,768 (42.1)	738 (41.0)
≥78	872 (14.5)	614 (14.6)	258 (14.3)
**Sex**			
Male	4,138 (68.9)	2,898 (69.0)	1,240 (68.8)
Female	1,867 (31.1)	1,305 (31.0)	562 (31.2)
**Race**			
White	4,937 (82.2)	3,485 (82.9)	1,452 (80.6)
Black	601 (10.0)	406 (9.7)	195 (10.8)
Other	467 (7.8)	312 (7.4)	155 (8.6)
**Histologic type**			
ccRCC	2,929 (48.8)	2,058 (49.0)	871 (48.3)
pRCC	268 (4.5)	173 (4.1)	95 (5.3)
sRCC	337 (5.6)	235 (5.6)	102 (5.7)
chRCC	41 (0.7)	30 (0.7)	11 (0.6)
CDC	43 (0.7)	34 (0.8)	9 (0.5)
RCC	2,387 (39.8)	1,673 (39.8)	714 (39.6)
**Size**	0.123		
≤ 82	2,772 (46.2)	1,946 (46.3)	826 (45.8)
83–140	2,622 (43.7)	1,835 (43.7)	787 (43.7)
≥141	611 (10.2)	422 (10.0)	189 (10.5)
**Grade**			
GI-II	682 (11.4)	477 (11.3)	205 (11.4)
GIII-IV	2,200 (36.6)	1,534 (36.5)	666 (37.0)
Unknown	3,123 (52.0)	2,192 (52.2)	931 (51.7)
***T*** **stage**			
*T*1	1,189 (19.8)	835 (19.9)	354 (19.6)
*T*2	1,231 (20.5)	847 (20.2)	384 (21.3)
*T*3	2,538 (42.3)	1,775 (42.2)	763 (42.3)
*T*4	605 (10.1)	426 (10.1)	179 (9.9)
TX	442 (7.4)	320 (7.6)	122 (6.8)
***N*** **stage**			
*N*0	3,934 (65.5)	2,789 (66.4)	1,145 (63.5)
*N*1	1,139 (19.0)	788 (18.7)	351 (19.5)
*N*2	932 (15.5)	626 (14.9)	306 (17.0)
**Bone metastasis**			
No	3,676 (61.2)	2,592 (61.7)	1,084 (60.2)
Yes	2,329 (38.8)	1,611 (38.3)	718 (39.8)
**Lung metastasis**			
No	2,294 (38.2)	1,618 (38.5)	676 (37.5)
Yes	3,711 (61.8)	2,585 (61.5)	1,126 (62.5)
**Liver metastasis**			
No	4,845 (80.7)	3,397 (80.8)	1,448 (80.4)
Yes	1,160 (19.3)	806 (19.2)	354 (19.6)
**Brain metastasis**			
No	5,299 (88.2)	3,732 (88.8)	1,567 (87.0)
Yes	706 (11.8)	471 (11.2)	235 (13.0)
**Surgery**			
No	3,383 (56.3)	2,356 (56.1)	1,027 (57.0)
Yes	2,622 (43.7)	1,847 (43.9)	775 (43.0)
**Radiotherapy**			
No	4,220 (70.3)	2,973 (70.7)	1,247 (69.2)
Yes	1,785 (29.7)	1,230 (29.3)	555 (30.8)
**Chemotherapy**			
No	2,918 (48.6)	2,084 (49.6)	834 (46.3)
Yes	3,087 (51.4)	2,119 (50.4)	968 (53.7)

*ccRCC, clear cell renal cell carcinoma; pRCC, papillary renal cell carcinoma; chRCC, chromophobe renal cell carcinoma; sRCC, sarcomatoid renal cell carcinoma; cdRCC, collecting duct renal cell carcinoma; RCC, renal cell carcinoma*.

### Identifying Independent Risk Factors

In the primary cohort, we identified the risk variables associated with early death of mRCC by utilizing univariate and multivariate logistic regression analyses (Table 3). Univariate logistic models displayed age at diagnosis, race, grade, *T*-stage, *N*-stage, histologic type, metastatic sites (bone, lung, liver and brain) and treatments (surgery, radiation, and chemotherapy) were associated with early death. Multivariate analysis revealed that 11 independent risk factors related to the early death of metastatic renal cell carcinoma including age, grade, *N*-stage, histologic type, metastatic sites (bone, lung, liver and brain) and treatments (surgery, radiation, and chemotherapy).

**Table 3:** Univariate and multivariate logistic regression for identifying the risk factors for early death of mRCC.

**Variable**	**Univariate analysis**			**Multivariate analysis**		
	**OR**	**95% CI**	***P*-value**	**OR**	**95% CI**	***P*-value**
**Age (years)**						
≤ 61	Ref			Ref		
62–77	1.668	1.437–1.935	<0.001*	1.388	1.159–1.664	<0.001*
≥78	3.474	2.864–4.213	<0.001*	1.672	1.306–2.140	<0.001*
**Sex**						
Male	Ref			-		
Female	1.192	1.036–1.372	0.014	-	-	-
**Race**						
White	Ref			Ref		
Black	1.337	1.079–1.656	**0.008**	1.008	0.773–1.314	0.954
Other	0.660	0.501–0.869	**0.003**	0.686	0.490–0.960	0.028
**Histologic type**						
ccRCC	Ref					
pRCC	1.332	0.910–1.950	0.140	1.202	0.773–1.868	0.414
sRCC	3.150	2.366–4.194	<0.001*	2.806	1.994–3.950	<0.001*
chRCC	0.350	0.083–1.475	0.153	0.357	0.075–1.693	0.195
CDC	3.031	1.504–6.112	**0.002**	2.910	1.279–6.623	**0.010**
RCC	4.371	3.762–5.077	<0.001*	1.954	1.616–2.362	<0.001*
**Size**						
≤ 82	Ref			-		
83–140	1.017	0.885–1.168	0.816	-	-	-
≥141	1.143	0.913–1.431	0.245	-	-	-
**Grade**						
GI-II	Ref			Ref		
GIII-IV	1.506	1.130–2.007	**0.005**	2.144	1.515–3.033	<0.001*
Unknown	4.360	3.322–5.722	<0.001*	1.388	0.996–1.935	0.053
***T*** **-stage**						
*T*1	Ref			Ref		
*T*2	0.953	0.776–1.170	0.645	1.087	0.844–1.400	0.519
*T*3	0.612	0.510–0.735	<0.001*	1.280	1.001–1.636	0.049
*T*4	1.540	1.210–1.959	<0.001*	1.739	1.284–2.357	<0.001*
TX	2.355	1.810–3.064	<0.001*	1.479	1.084–2.017	0.014
***N*** **stage**						
*N*0	Ref			Ref		
*N*1	1.717	1.454–2.028	<0.001*	1.365	1.110–1.680	**0.003**
*N*2	1.707	1.423–2.047	<0.001*	1.850	1.471–2.327	<0.001*
**Bone metastasis**						
No	Ref			Ref		
Yes	1.097	0.959–1.255	**0.177**	1.316	1.088–1.591	**0.005**
**Lung metastasis**						
No	Ref			Ref		
Yes	1.300	1.133–1.490	<0.001*	1.387	1.163–1.653	<0.001*
**Liver metastasis**						
No	Ref			Ref		
Yes	2.599	2.219–3.043	<0.001*	1.989	1.635–2.419	<0.001*
**Brain metastasis**						
No	Ref			Ref		
Yes	1.685	1.384–2.051	<0.001*	2.115	1.618–2.765	<0.001*
**Surgery**						
No	Ref			Ref		
Yes	0.146	0.124–0.173	<0.001*	0.151	0.116–0.198	<0.001*
**Radiotherapy**						
No	Ref			Ref		
Yes	0.666	0.573–0.774	<0.001*	0.651	0.521–0.813	<0.001*
**Chemotherapy**						
No	Ref			Ref		
Yes	0.218	0.189–0.252	<0.001*	0.175	0.147–0.209	<0.001*

*ccRCC, clear cell renal cell carcinoma; pRCC, papillary renal cell carcinoma; chRCC, chromophobe renal cell carcinoma; sRCC, sarcomatoid renal cell carcinoma; cdRCC, collecting duct renal cell carcinoma; RCC, renal cell carcinoma*.

*The bold values mean statistically significance (p < 0.01)*.

### Nomogram Construction

**Figure 3:**
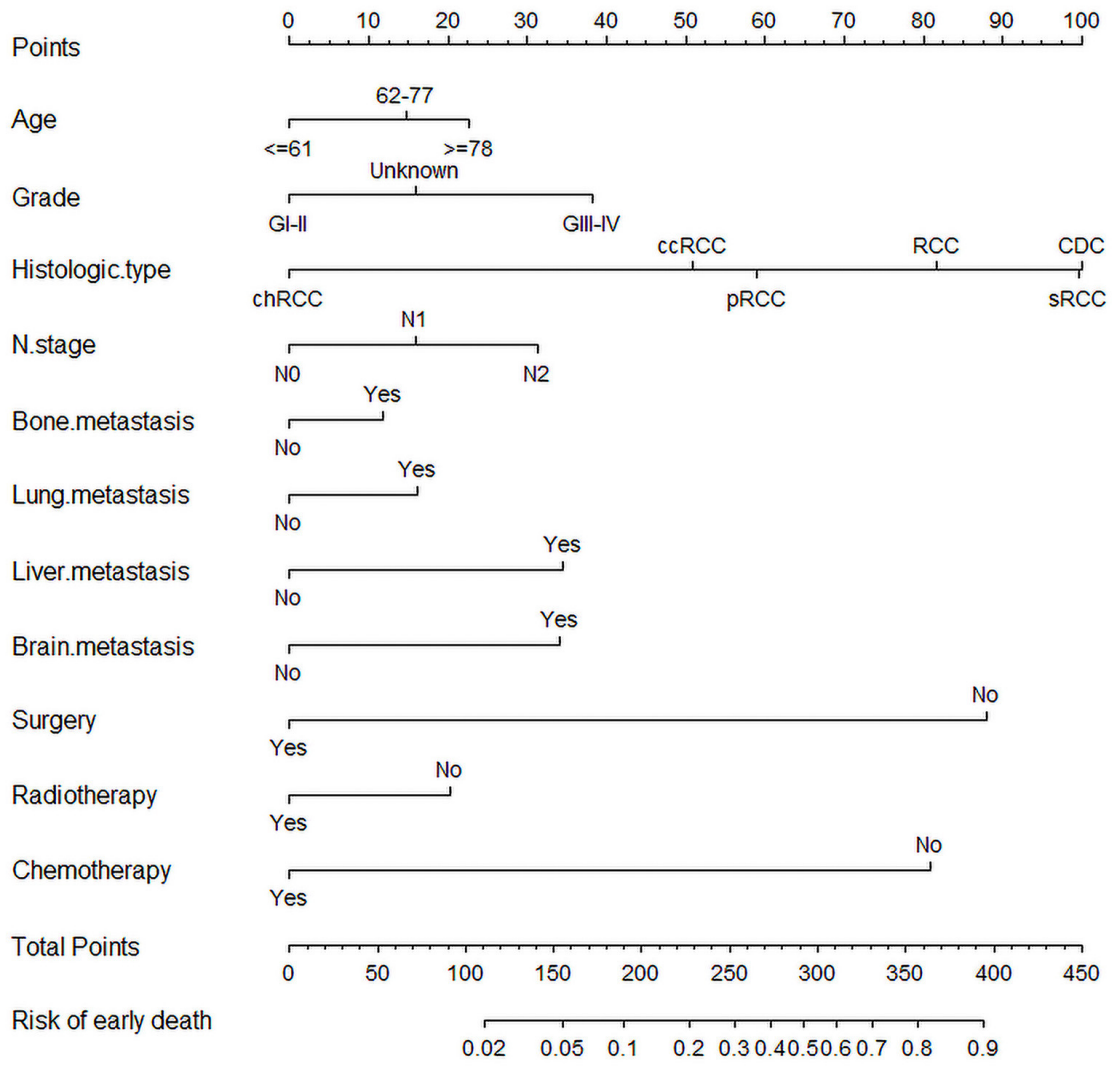
The nomogram for predicting early death in metastatic renal cell carcinoma.

Significant and independent risk factors from multiple logistic regression were acquired to construct a comprehensive nomogram for predicting early death in mRCC (Figure 3). In the prediction model, surgery, chemotherapy and histological classification had great predictive value. We can predict the odds of early death of mRCC by calculating the sum of the scores of each variable.

### Performance of Nomograms

**Figure 4:**
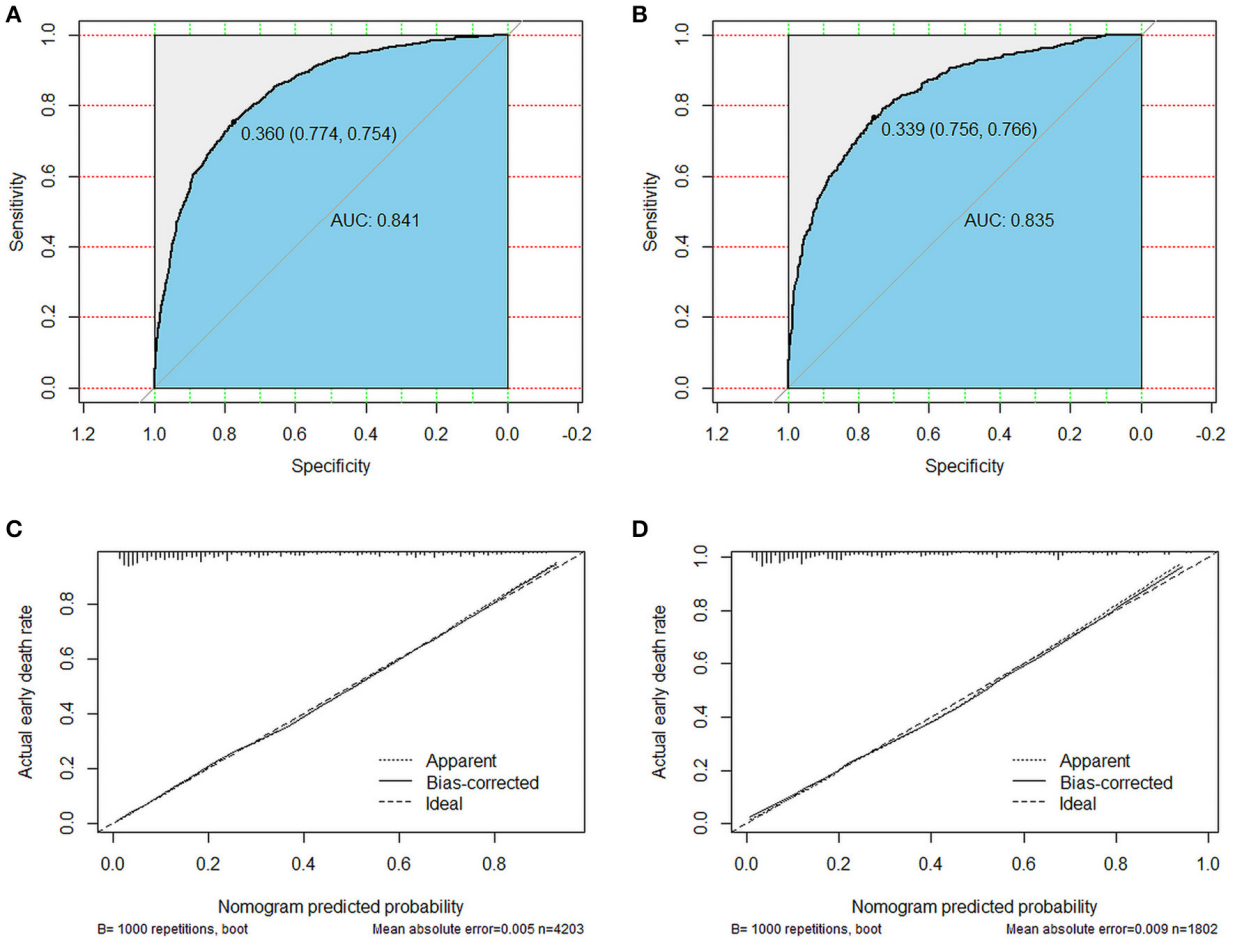
ROC curves and calibration plots for the nomogram. The ROC curves for the nomogram in the training cohort **(A)** and the validation cohort **(B)**. The calibration plots for the nomogram in the training cohort **(C)** and the validation cohort **(D)**.

**Figure 5:**
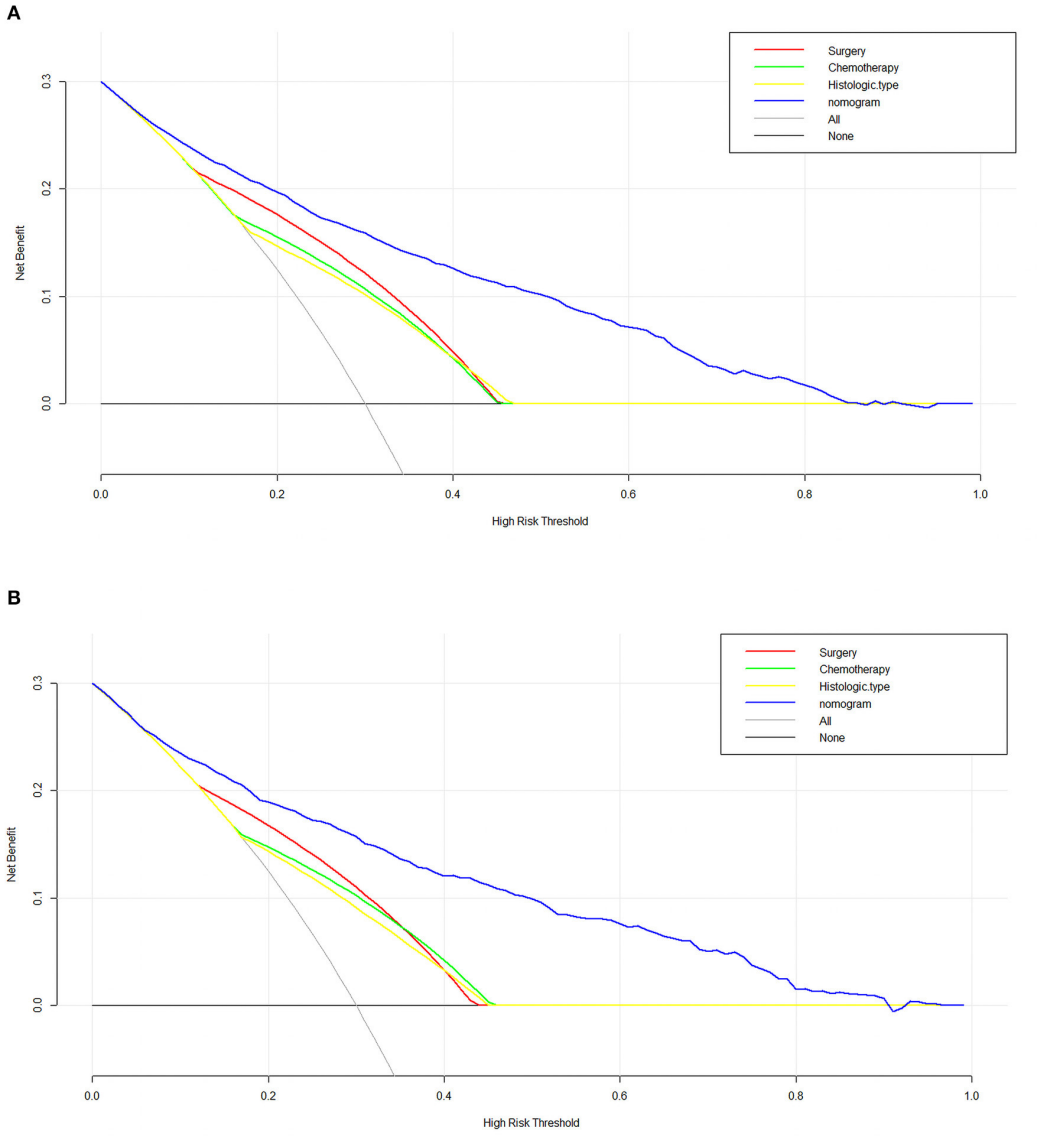
The decision curve analysis (DCA) curve for the nomogram in the training cohort **(A)** and the Validation cohort **(B)**.

In order to test our predictive model, we applied the C-index, AUC and calibration curves. The C-index of 0.842 for the predictive nomogram was detected in the primary cohort, and similar C-index was 0.863 in the validation cohort. In the primary and validation cohort, the AUC values were 0.841 (95% CI 0.828–0.854) and 0.835 (95% CI 0.814–0.855), respectively, which means an excellent discrimination capability in predicting early death of metastatic renal cell carcinoma (Figures 4A,B). Moreover, whether in the training or validation set, the solid lines of the calibration curves of both are close to 45°, which suggested the model was reliable (Figures 4C,D). Decision curve analysis (DCA) as an advanced method could evaluate the clinical efficacy of the nomogram. Our results suggested that there were excellent net benefits among most of the threshold probabilities, both in the primary cohort and in the validation cohort (Figure 5).

## Discussion

As the most common renal cancer, renal cell carcinoma causes a bad influence among over 400,000 individuals worldwide per year (14). Although the treatment of mRCC has made progress in the past two decades, the prognosis of patients with mRCC remains dismal (15). Previous studies on mRCC generally concentrated on the long-term survival of patients (16, 17) or risk factors related to lung and bone metastasis (18, 19). However, advanced or highly invasive tumors often lead to early death and few study has identified risk factors associated with early death in mRCC. In 2019, Shin et al. (20) constructed a model for predicting early death within 1 year in patients with mRCC after first-line tyrosine kinase inhibitors (TKIs) administration. In our study, we defined it as survival ≤ 3 months according to the previous definition of early death (10, 21). The early mortality rates from all cause and cancer-specific cause for metastatic renal cell carcinoma were 30.2 and 28.1%, respectively. In addition, our subjects were not only patients treated with TKIs, but all patients with mRCC, and more patients and risk factors were included. Exploring risk factors for early death in mRCC is beneficial, which can assist clinicians to formulate individualized treatment plans and carry out clinical trials. In addition, it is also conducive to reduce burden on patients. Because for some specific patients, they can't benefit from treatment. Surgery may bring many side effects such as cardiovascular and cerebrovascular accidents and it is extraordinary inconvenient to go to the hospital. Consequently, we established a predictive nomogram for recognizing the early death of mRCC patients.

Previous studies had reported that demographic information such as diagnostic age, gender and race were explored to be closely associated with renal cell carcinoma (22–24). The impact of such demographic factors on early death of mRCC were evaluated in our model. But the results displayed that only age made a difference. In addition to these demographic factors, the early death of mRCC was mostly connected with clinical factors including tumor stage, histological classification, *N*-stage, metastatic status and treatment (surgery, chemotherapy and radiotherapy). In the era of targeted therapy for renal cell carcinoma, it is controversial whether cytoreductive nephrectomy and complete metastasectomy can bring survival benefits. In a randomized trial (CARMENA), sunitinib monotherapy was not inferior to sunitinib in the treatment of moderate to low-risk metastatic RCC after nephrectomy, which was supported by most scholars (25). However, the trial was based on MSKCC model, lacking some other relevant factors. Several retrospective studies have shown that patients with mRCC receiving targeted therapy can obtain survival benefits from nephrectomy (26–28). These analyses were limited by the nature of retrospective analysis. It should be noted that in any case, the choice of patients and the timing of surgery cause a great influence on the benefits of nephrectomy in patients with mRCC (25, 27). For patients in good condition, surgery can significantly reduce tumor burden and prolong overall survival (29). Collecting duct renal cell carcinoma (cdRCC) and sarcomatoid renal cell carcinoma (sRCC) were associated with an aggressive biology and characterized by a poor prognosis (30, 31). Consistent with previous studies, histological classification such as cdRCC and sRCC was significantly related to early death of mRCC. Metastatic renal cell carcionoma with lymph node metastasis and high-level pathological grade possessed highly aggressive and invasive characteristics, which had a negative impact on the survival of patients with mRCC (17, 32). These patients were often prone to premature death. A result from the multi-institutional registry (REMARCC) showed that different metastatic sites had different effects on the survival benefits of patients with mRCC. The survival rate of patients with distant metastasis were often worse (33). Similar to their study, we found that patients with distant metastasis were often apt to early death, especially those with liver and brain metastasis.

The SEER database was employed in our nomogram. Thus, our analysis was based on large sample sizes, which ensured the reliability of our results. By completing curve analysis and internal verification, our model showed an excellent performance in respect of accuracy and discrimination. In addition, our nomogram was characterized by clinical practicality. As an advanced tool, DCA is different from traditional ROC analysis and can be applied to examine whether model-based clinical decisions are effective (34). Our study displayed the net benefit of our nomogram was better than that in other two scenarios (all screening or none-screening) between 10 and 90%.

Nevertheless, there were several inevitable limitations in this study that require consideration. First of all, some known relative factors were not taken into account in the nomogram. For example, the comorbidities and performance status and the Fürhman classification are thought to be related to mRCC prognosis. In addition, the number of distant metastases at diagnosis was not explored in our study, the prognosis of mRCC patients with multiple metastases was generally poor. Secondly, our study was developed retrospectively and potential selection bias may adversely affect the conclusion. Thirdly, the detailed information on chemotherapy and specific surgical procedures were lacking in the SEER database. Fourthly, although our model was validated internally, it is necessary to carry out external verification. In the future, wo need to combine with other research data to predict the early death of mRCC.

## Conclusion

In conclusion, a comprehensive nomogram for predicting early death in metastatic renal cell carcinoma was developed according to 11 significant risk factors distinguished by univariate and multivariate logistic analysis. This nomogram is conducive for surgeons to formulate targeted treatment strategies and improve survival outcomes for patients with metastatic renal cell carcinoma.

## Data Availability Statement

Publicly available datasets were analyzed in this study. This data can be found here: https://seer.cancer.gov/data/.

## Author Contributions

TC and XZhan formulated the study. XZhan and XZhang explored and analyzed the datal. YX, SZ, and MJ finished the manuscript. LC and BF proofread the manuscript. All authors approved the final version. All authors contributed to the article and approved the submitted version.

## Funding

This work was funded by the National Natural Science Foundation of P.R. China (Grant Nos. 81560419, 81960512, and 81760457) and Jiangxi Provincial Double Thousand Plan Fund Project (Grant No. jxsq2019201027).

## Conflict of Interest

The authors declare that the research was conducted in the absence of any commercial or financial relationships that could be construed as a potential conflict of interest.

## Publisher's Note

All claims expressed in this article are solely those of the authors and do not necessarily represent those of their affiliated organizations, or those of the publisher, the editors and the reviewers. Any product that may be evaluated in this article, or claim that may be made by its manufacturer, is not guaranteed or endorsed by the publisher.
